# Paradoxical lichenoid reaction during dupilumab treatment- a case report and literature review^؆^

**DOI:** 10.1016/j.abd.2025.501192

**Published:** 2025-08-22

**Authors:** Fuchen Huang, Zequn Tong, Xueting Zeng, Jiawen Chen, Ying Zou, Chao Ji

**Affiliations:** a Department of Dermatology, First Affiliated Hospital, Fujian Medical University, Fuzhou, Fujian, China; b Fujian Dermatology and Venereology Research Institute, First Affiliated Hospital, Fujian Medical University, Fuzhou, Fujian, China; c Key Laboratory of Skin Cancer, Fujian Higher Education Institutions, Fuzhou, Fujian, China

Dear Editor,

Paradoxical reactions (PRs) are defined as the onset of new or the exacerbation of existing immune-mediated disorders following the initiation of targeted biologic therapies.^1^, ^2^ Dupilumab, an IL-4Rα inhibitor, is effective for atopic dermatitis (AD) but may trigger PRs. While paradoxical psoriasiform or head and neck dermatitis are common, lichenoid reactions remain rare.^2^ We present a case of dupilumab-induced paradoxical lichenoid reaction with erythroderma and review existing literature to identify clinical patterns.

A 64-year-old male with refractory AD developed erythroderma 4 months after initiating dupilumab therapy. He reported improved pruritus and lesions after the first dose of dupilumab (600 mg). However, he began developing pruritic scaly erythematous patches since the second dose of dupilumab (300 mg every other week), which gradually progressed to erythroderma and hyperpigmentation. Physical examination revealed diffuse erythema, scaling, and symmetrical violaceous papules on the dorsal hands (Fig. 1A‒C). Histopathology demonstrated lichenoid dermatitis, featuring parakeratosis, necrotic keratinocytes, and a band-like dermal infiltrate of lymphocytes and histiocytes (Fig. 2A‒C). Immunohistochemistry (IHC) confirmed the presence of T-cell (Fig. 3A‒B; CD4 and CD8; CD4/CD8 ratio of 3:2) and histiocytic proliferation (Fig. 3C; CD68). IHC staining also confirmed the expression of CD3, CD5, and CD7, while showing negativity for CD20, perforin, and granzyme B. Few cells were positive for Langerin. Dupilumab was discontinued after the fifth dose. Treatment with oral methylprednisolone, upadacitinib, antihistamines, and topical steroids led to erythema resolution within 3-weeks, though hyperpigmentation persisted. At 3-month follow-up, no disease flares occurred, and pruritus remained controlled. Concomitant medications (betastin, cetirizine) had been used long-term without prior adverse effects, supporting dupilumab as the likely trigger. This case highlights dupilumab-induced lichenoid reaction manifesting as erythroderma, necessitating histopathological confirmation for timely intervention.

**Fig. 1:**
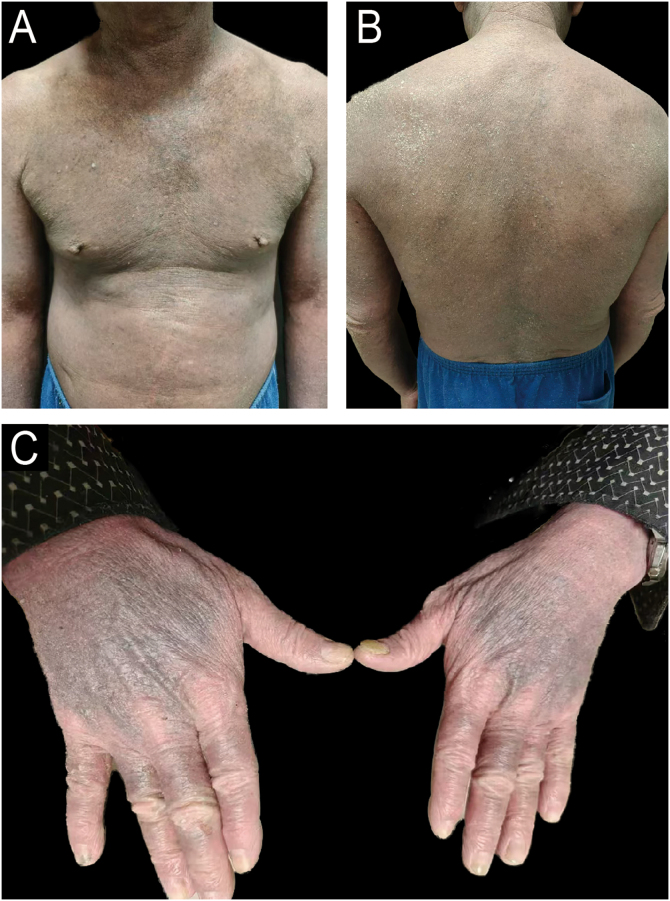
(A‒B) The patient presented to our hospital with generalized erythema, hyperpigmentation and scaling over the entire body. (C) Symmetrical flat-topped violaceous papules were observed on the dorsal hands.

**Fig. 2:**
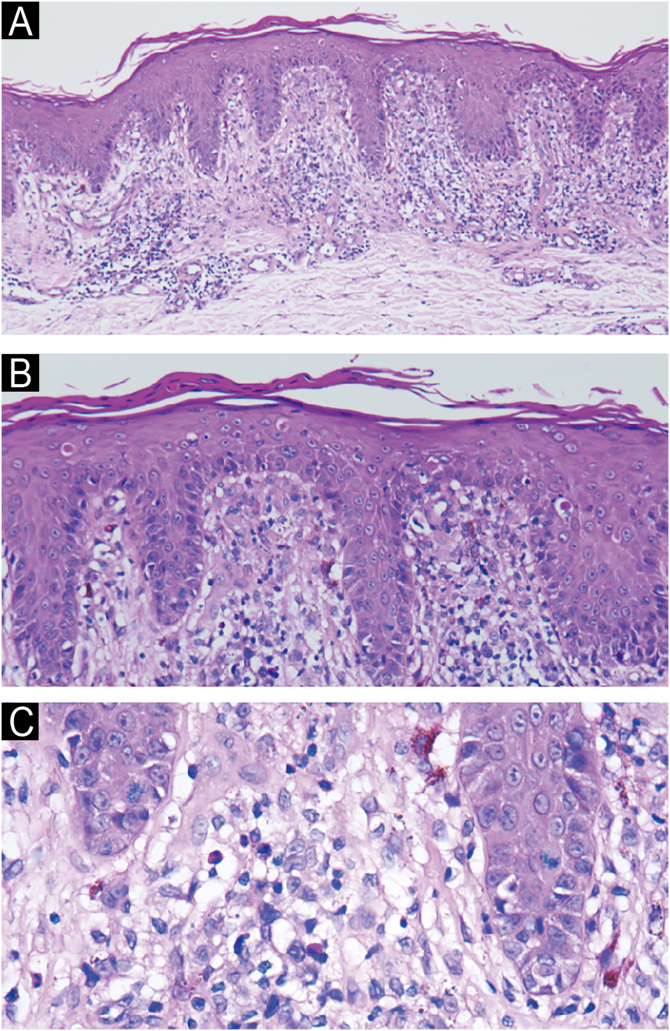
(A‒C) Histopathology revealed epidermal parakeratosis, acanthosis, scattered necrotic keratinocytes and basilar vacuolar change. A band-like infiltrate of lymphocytes, histiocytes, melanophages, eosinophils, and neutrophils was present at the dermoepidermal junction. (A, Hematoxylin & eosin ×10; B, Hematoxylin & eosin ×20; C, Hematoxylin & eosin ×40).

**Fig. 3:**
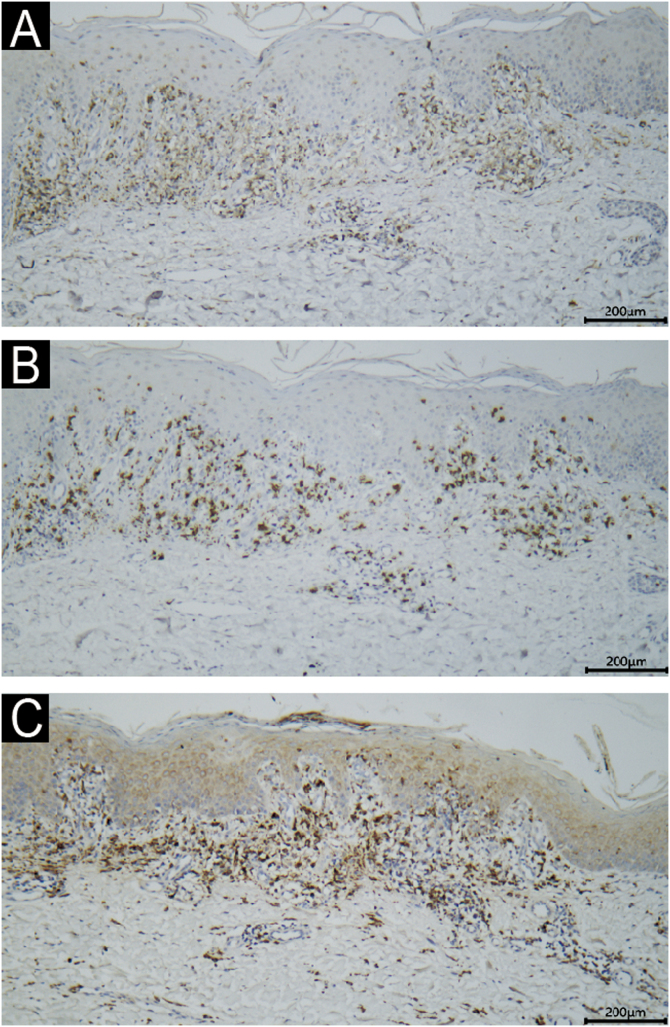
Immunohistochemical staining demonstrated that the lymphocytes stained positive for CD4 (A, ×10) and CD8 (B, ×10), with a CD4/CD8 ratio of approximately 3:2. The histiocytes stained positive for CD68 (C, ×10).

A review of seven reported cases of dupilumab-associated lichenoid reactions was conducted (Table 1).^3^, ^4^, ^5^, ^6^, ^7^ Patients predominantly affected were female (71.4%, 5/7) with a mean age of 45.3 years, all diagnosed with AD. The median latency from dupilumab initiation to lichenoid reactions was 29.7 weeks. Clinically, 85.7% (6/7) presented with lichen planus (LP) like violaceous papules or plaques, while 28.6% (2/7) developed erythroderma. Lesions primarily involved the hands and extremities (85.7%) and trunk (57.1%), with oral mucosa involvement in 28.6%. Histopathology categorized lichenoid reactions into three subtypes: classic LP-type (33%), lichenoid drug eruption (LDE)-type (33%), and lichenoid granulomatous reaction (LGR)-type (33%), the latter characterized by granulomatous inflammation with eosinophils and histiocytes. The histopathological findings in this case are consistent with LDE-type, characterized by lichenoid interface dermatitis accompanied by parakeratosis and a mixed infiltration of lymphocytes, histiocytes, and eosinophils. Management universally included dupilumab discontinuation (85.7%), with systemic therapies such as glucocorticoids (57.1%), JAK inhibitors (28.6%), and cyclosporine or methotrexate (14.3%) employed. Resolution occurred within a median of 15.8 weeks post-treatment. Notably, two cases exhibited persistent post-inflammatory hyperpigmentation.^4^ These findings underscore the variability in dupilumab-induced lichenoid reactions, emphasizing the need for early recognition via biopsy and tailored immunomodulatory strategies.

**Table 1:** Cases of lichenoid reactions associated with dupilumab therapy.

Author (year)	Age/ Sex	Primary disease	Latent period	Clinical manifestations	Distribution patterns	Histopathology	Diagnosis	Treatment for lichenoid reaction	Resolution time	Treatment response
Tae-Eun et al. (2021) ^3^	44, M	AD	4 months	Localized flat-topped erythematous to violaceous papules and plaques	Dorsum of both hands	LP type	Lichenoid reaction	Cessation of dupilumab and cyclosporin	3 months after dupilumab cessation	Partial response
Laura et al. (2022) ^4^	23, F	AD	11 months	Flat-topped violaceous papules	Wrists, fingers, abdominal wall, upper thighs, lower legs and oral mucosa	LP type	Drug-induced lichen planus	Cessation of dupilumab and systematic glucorticosteroids	4 months after dupilumab cessation	Marked improvement with hyperpigmentation
Mark et al. (2022) ^5^	18, F	AD	14 months	Erythematous plaques with scale	Chest and abdomen	LDE type	Lichenoid reaction	Cessation of dupilumab and upadacitinib	NA	Na
Luca et al. (2022) ^6^	60 s, F	AD	More than a year	Papular, polygonal, confluent, very itchy lesions	Upper and lower extremities	NA	Lichen ruber planus	Continuation of dupilumab, systematic glucorticosteroids and topical glucorticosteroids	4 months after treatment	Complete resolution with residual lichenification and xerosis
Zahra et al. (2023) ^7^	72, F	AD	1) 15 months; 2) 24 months; 3) 28 months	1) Erosive lichenoid mucositis; 2) Violaceous, indurated plaques; 3) Erythroderma	1) Oral mucosa; 2) Widespread; 3) Widespread, including hands	LGR type	Lichenoid granulomatous drug reaction	1) Continuation of dupilumab, systematic glucorticosteroids and alitretinoin; 2) Continuation of dupilumab, systematic glucorticosteroids and topical glucorticosteroids; 3) Acitretin with no improvement, methotrexate, and cessation of dupilumab	6 months after dupilumab cessation	Complete clearance
	51, F	AD	15 months	Erythematous and indurated plaques with lichenification and fissuring	Predominantly hands	LGR type	Lichenoid granulomatous drug reaction	Cessation of dupilumab and methotrexate	A period of months after dupilumab cessation	Complete clearance
Present case	64, M	AD	2 weeks	Pruritic scaly erythematous plaques progressing to erythroderma; and violaceous papules	Widespread and dorsum of both hands	LDE type	Lichenoid reaction	Cessation of dupilumab, systematic glucorticosteroids, antihistamines, topical steroids and upadacitinib	11 weeks after dupilumab cessation	Complete resolution with hyperpigmentation

F, Female; M, Male; AD, Atopic Dermatitis; LP, Lichen Planus; LDE, Lichenoid Drug Eruption; LGR, Lichenoid Granulomatous reaction; NA, Not Available.

This case highlights dupilumab-induced lichenoid reaction presenting as erythroderma. This patient was initially misdiagnosed as an exacerbation of AD, leading to the continued administration of dupilumab, which further worsened the patient's symptoms. However, subsequent histological examination confirmed the diagnosis of paradoxical lichenoid reaction, enabling the appropriate treatment and subsequent disease remission. Histopathology and immunohistochemistry revealed CD8^+^ T-cell infiltration and histiocytic proliferation, aligning with a delayed hypersensitivity reaction.^8^ The absence of perforin and granzyme-B suggests alternative pathways beyond cytotoxic T-cell activity, possibly involving Th1/Th2 imbalance. The downregulation of the Th2 response characteristic of AD could promote a Th1 response by increasing Th1 cell activity and IFN-γ production due to IL-4 antagonism.^3^

These findings emphasize the need for heightened vigilance regarding paradoxical reactions during biologic therapies. In addition, AD is associated with higher risks of Cutaneous T-Cell Lymphoma (CTCL), which could also mimic eczematous skin lesions or erythroderma, requiring skin biopsy for accurate diagnosis.^9^ Clinically, violaceous plaques or sudden erythroderma in dupilumab-treated patients warrant immediate biopsy to differentiate other conditions, including lichenoid reaction and CTCL from AD exacerbation. Continued dupilumab administration in misdiagnosed cases risks worsening symptoms, as observed here. Discontinuation of dupilumab, combined with systemic steroids or JAK inhibitors (e.g., upadacitinib), achieved rapid remission in this and prior cases, though post-inflammatory hyperpigmentation may persist. While JAK inhibitors show promise in managing PRs, further research is required to elucidate precise molecular mechanisms and optimize therapeutic strategies.^10^

## Research data availability

Does not apply.

## Scientific associate editor

Hiram Larangeira de Almeida Jr.

## Financial support

None declared.

## Authors’ contributions

Fuchen Huang: Conception and design of the study; data collection, or analysis and interpretation of data; statistical analysis; writing of the manuscript; data acquisition, analysis, and interpretation; critical review of the literature.

Zequn Tong: Analysis and interpretation of data; critical revision of the manuscript for important intellectual content.

Xueting Zeng: Analysis and interpretation of data; critical revision of the manuscript for important intellectual content.

Jiawen Chen: Analysis and interpretation of data; critical revision of the manuscript for important intellectual content.

Ying Zou: Critical revision of the manuscript for important intellectual content.

Chao Ji: Significant participation in research supervision; critical revision of the manuscript for important intellectual content.

## Conflicts of interest

None declared.
